# Association between Ghrelin gene (GHRL) polymorphisms and clinical response to atypical antipsychotic drugs in Han Chinese schizophrenia patients

**DOI:** 10.1186/1744-9081-8-11

**Published:** 2012-02-28

**Authors:** Yongfeng Yang, Wenqiang Li, Jingyuan Zhao, Hongxing Zhang, Xueqin Song, Bo Xiao, Ge Yang, Chengdi Jiang, Dai Zhang, Weihua Yue, Luxian Lv

**Affiliations:** 1 Department of Psychiatry, The Second Affiliated Hospital of Xinxiang Medical University, Xinxiang, China; 2 Henan Mental Hospital, Henan Key Lab of Biological Psychiatry, Xinxiang, China; 3 First Affiliated Hospital of Zhengzhou University, Zhengzhou, China; 4 Key Laboratory for Mental Health, Ministry of Health, Institute of Mental Health, Peking University, Beijing, China

**Keywords:** Schizophrenia, Ghrelin (*GHRL*), Polymorphrism, Body mass index (BMI), Atypical antipsychotics, Therapeutic effects

## Abstract

**Background:**

Ghrelin (*GHRL*) is a pivotal peptide regulator of food intake, energy balance, and body mass. Weight gain (WG) is a common side effect of the atypical antipsychotics (AAPs) used to treat schizophrenia (SZ). Ghrelin polymorphisms have been associated with pathogenic variations in plasma lipid concentrations, blood pressure, plasma glucose, and body mass index (BMI). However, it is unclear whether *GHRL *polymorphisms are associated with WG due to AAPs. Furthermore, there is no evidence of an association between *GHRL *polymorphisms and SZ or the therapeutic response to AAPs. We explored these potential associations by genotyping *GHRL *alleles in SZ patients and controls. We also examined the relation between these SNPs and changes in metabolic indices during AAP treatment in SZ subgroups distinguished by high or low therapeutic response.

**Methods:**

Four SNPs (Leu72Met, -501A/C, -604 G/A, and -1062 G > C) were genotyped in 634 schizophrenia patients and 606 control subjects.

**Results:**

There were no significant differences in allele frequencies, genotype distributions, or the distributions of two SNP haplotypes between SZ patients and healthy controls (*P *> 0.05). There was also no significant difference in symptom reduction between genotypes after 8 weeks of AAP treatment as measured by positive and negative symptom scale scores (PANSS). However, the -604 G/A polymorphism was associated with a greater BMI increase in response to AAP administration in both APP responders and non-responders as distinguished by PANSS score reduction (*P *< 0.001). There were also significant differences in WG when the responder group was further subdivided according to the specific AAP prescribed (*P *< 0.05).

**Conclusions:**

These four *GHRL *gene SNPs were not associated with SZ in this Chinese Han population. The -604 G/A polymorphism was associated with significant BW and BMI increases during AAP treatment. Patients exhibiting higher WG showed greater improvements in positive and negative symptoms than patients exhibiting lower weight gain or weight loss.

## Background

Schizophrenia (SZ) is a severe brain disorder afflicting approximately 1% of the world's population and often leads to a lifetime of disability and emotional distress [1]. Family, twin, and adoption studies strongly indicate that genetics contribute to the etiology of SZ, probably by transmission of multiple susceptibility genes each exerting weak-to-moderate effects on predisposition [2,3]. Many candidate susceptibility genes have been identified, including the dopamine receptor D2, neuregulin1, and disrupted in schizophrenia 1 (DISC-1) [4-6].

Epidemiological studies have also revealed that people with SZ are at greater risk for obesity, type 2 diabetes, dyslipidemia, and hypertension than the general population [7]. Recently, it was suggested that SZ patients are at increased risk of metabolic problems and that the associated symptoms are a serious threat to patient health [8]. Metabolic problems are often triggered by antipsychotic medication. Indeed, significant weight gain is common in AAP-treated SZ patients, especially patients administered clozapine, olanzapine, quetiapine, or risperidone [9].

The peptide ghrelin (product of the *GHRL *gene) is an important metabolic regulator produced by the stomach and pancreas. Specific SNPs of *GHRL *have been associated with variations in BMI, blood pressure, high-density lipoproteins, low-density lipoproteins, serum cholesterol, blood glucose, and metabolic syndrome [10-14]. Ghrelin, originally isolated from the rat stomach, stimulates food intake and controls energy balance [15,16]. Studies on animal models revealed that *GHRL *increased food intake and adiposity [17,18]. However, circulating *GHRL *levels were decreased in obese individuals, and serum *GHRL *levels were inversely correlated with BMI, suggesting that *GHRL *is not directly involved in most cases of obesity [19,20]. Studies on the relationship between WG, circulating *GHRL*, and AAP have yielded inconsistent findings. Patients taking clozapine or olanzapine showed greater WG than patients on other antipsychotics [21]. In one study, plasma total *GHRL *and active *GHRL *were increased significantly immediately after olanzapine treatment, but the changes in BMI and body weight were not significant after 6 months of treatment [22]. In contrast, another study found that serum bioactive *GHRL *levels decreased significantly from baseline after 4 weeks of olanzapine monotherapy [23]. In humans, *GHRL *plays an important role in the long-term regulation of body weight (BW) as well as in the short-term regulation of appetite [19,21]. Ghrelin stimulated preadipocyte differentiation, increased the BMI, and inhibited the anorexigenic effect of leptin [24]. Drug altering *GHRL *function may have distinct short- and long-term effects on BMI.

The human *GHRL *gene is located on chromosome 3 (3p25-p26), and consists of 4 exons and 3 introns [25,26]. Several SNPs in the coding region of prepro-ghrelin have been described, but there is no known specific association between genetic variations in the human *GHRL *gene and SZ risk. However, region 3p25.1-26.1 is strongly associated with schizophrenia. In addition to *GHRL*, this region contains SYN2, HRH1, and GRM7, all candidate genes for schizophrenia. The positive symptoms of schizophrenia are associated with dysfunction in dopaminergic signaling, which is closely associated with a *GHRL *mutation [27-29]. Previous studies demonstrated that SNPs in *GHRL *were associated with high BMI; the Leu72Met allele was significantly associated with BMI and coronary artery disease [18,19,30], but this was not confirmed in other studies [26,31,32].

In light of the increase metabolic syndrome symptoms observed in SZ patients on AAPs, as well as the important role of *GHRL *as a metabolic regulator and the association between *GHRL *SNPs and metabolic indices, we hypothesized that (1) *GHRL *might be a candidate gene for SZ and that (2) allelic variants of *GHRL *might be associated with the propensity for BMI changes induced by AAP treatment. In addition, we tested (3) whether a putative relationship between *GHRL *SNPs and metabolic effects was specific to individual AAP types. Finally, (4) we examined if *GHRL *alleles influenced the clinical efficacy of AAPs. To these ends, we genotyped four SNPs and investigated whether they were associated with SZ and the therapeutic and metabolic effects of AAPs in the Han Chinese population.

## Methods

The study group consisted of 634 diagnosed schizophrenic patients (332 males and 302 females; mean age: 27.14 ± 7.53 years). Patients were unrelated Han Chinese born and living in the North Henan province, and all their biological grandparents were of Han Chinese ancestry. Individuals with a history of severe medical complications, organic brain disease, any concomitant major psychiatric disorders, or substance dependence were excluded. All patients were recruited from the Department of Psychiatry of the Second Affiliated Hospital of Xinxiang Medical University, P.R. China. The consensus diagnoses were conducted by at least two experienced psychiatrists according to the Diagnostic and Statistical Manual of Mental Disorders, fourth Edition (DSM-IV) [33]. The patient group included paranoid (n = 309), catatonic (n = 50), collapse (n = 45), residual (67), and undifferentiated (n = 163) schizophrenic types.

The control group consisted of 606 healthy subjects (293 males and 313 females; mean age: 29.08 ± 7.80 years) recruited from communities and colleges within the same region and matched to the patient group for age, gender ratio, and Han ethnicity. Controls were recruited using a simple non-structured interview performed by psychiatrists. Individuals with personal or family histories of mental illness or neurological diseases were excluded. The objectives and procedures of the study were explained to all subjects and written informed consent was obtained. The Ethical Committee of the Department of Psychiatry of the Second Affiliated Hospital of Xinxiang Medical University approved this study.

Three hundred and eighty patients were evaluated using the Positive and Negative Symptom Scale (PANSS) [34] before and after an 8-week administration of antipsychotic medications. Only those patients with total PANSS scores ≥ 60 before treatment were included. The reduction in PANSS scores from baseline after the 8-week treatment regime was used to evaluate the efficacy of each AAP. Patients were divided into 2 groups based on the reduction in PANSS score, a responder group exhibiting a > 50% reduction and none-responder group exhibiting a ≤ 50% reduction [35].

We excluded patients with incomplete clinical data. A total of 569 patients were treated by monotherapy using an AAP not previously prescribed. Patients were treated with clozapine (n = 103, 100-700 mg/d), risperidone (n = 181, 2-6 mg/d), olanzapine (n = 60, 5-20 mg/d), quetiapine (n = 126, 400-750 mg/d), ziprasidone (n = 61, 80-160 mg/d), or aripiprazole (n = 38, 10-30 mg/d). Body weight and BMI was measured before and after 4 weeks of AAP treatment. Individual BMIs were calculated as BMI = weight (kg)/height^2 ^(m).

Peripheral blood samples were obtained from the subjects and genomic DNA was prepared using the QIAamp DNA blood Mini Kit (QIAGEN, Hilden, Germany). Four SNPs [rs696217 (Leu72Met), rs26802 (-501A/C), rs27647 (-604 G/A), and rs26311 (-1062 G > C)] were selected according to the dbSNP database http://www.ncbi.nlm.nih.gov/SNP/. The SNPs rs27647 and rs26311 are located in the promoter region, rs26802 in intron 1, and rs696217 in exon 3 of *GHRL*. All these SNPs effect *GHRL *function and have been linked to metabolic symptoms. The rs696217 amino-acid change (Leu72Met) affects the tail of the pro-ghrelin molecule, but it is not known how this affects *GHRL *expression or activity (Table 1). The four SNPs were detected by polymerase chain reaction (PCR)-based restriction fragment length polymorphism (PCR-RFLP) analysis.

**Table 1:** SNPs and primers of PCRs and corresponding restriction enzymes

Marker	Location	Primer sequence (5'-3')	Product (bp)	Annealing temperature (°C)	RFLP	Allele (bp)
rs27647	Promoter	5'-CACAGCAACAAAGCTGCACC-3'	929	65	*Dra *I	A(929)
		5'-AAGTCCAGCCAGAGCATGCC-3'				G(664,265)
rs26802	Intron 1	5'-AGAACAAACGCCAGTCATCC-3',	205	55	*Mwo *I	A(205)
		5'-GTCTTCCAGCCAGACAGTCC-3'				C(104,101)
rs696217	Exon 3	5'-GCTGGGCTCCTACCTGAGC-3'	618	65	*Bsr *I	T(618)
		5'-GGACCCTGTTCACTGCCAC-3'				G(517,101)
rs26311	Promoter	5'-GGCAGCAGTCACGGACAATAAA-3'	779	55	*Bcn *I	G (572,252)
		5'-CTCAGAAGAGGCATCCGCTAAA-3'				C(527,191,61)

The primers of the four SNPs investigated are shown in Table 1. The conditions used for PCR amplification included an initial denaturation step at 94°C for 5 min, followed by 36 cycles of 94°C for 30 s, 55-65°C for 30 s, and 72°C for 1 min, followed by a final extension at 72°C for 10 min. Small volumes (10 μl) of these PCR products were completely digested with 2U of restriction enzyme (*Dra I *for -604 G/A, *Mwo I *for -501A/C, *Bsr I *for Leu72Met, and *Bcn I *for -1062 G > C). The fragments were separated on 2-4% agarose gels and visualized under ultraviolet light after staining with ethidium bromide.

The statistical power of the sample size was calculated by the genetic power calculator (GPC, http://pngu.mgh.harvard.edu/~purcell/gpc/cc2.html) [36]. Deviations in the genotype counts from Hardy-Weinberg equilibrium were tested using a *χ^2 ^*goodness-of-fit test. Statistical differences in genotypic, allelic, and haplotypic distributions between SZ and control subjects were evaluated by the *χ^2 ^*test with a significance level of 0.05. Odds ratios (OR) and 95% confidence intervals (95% CI) were calculated to evaluate the effects of different alleles on SZ risk. Pair-wise linkage disequilibrium (LD) analysis was applied to detect the inter-marker relationship using *D' *and *r^2 ^*values. Case-control association analysis was performed by SHEsis software http://analysis.bio-x.cn/myAnalysis.php[37], a powerful software platform for analyses of LD, haplotype construction, and genetic association at polymorphic loci. Associations between response to a specific AAP and genotype were determined by *t*-tests and analysis of variance (ANOVA) tests using SPSS 13.0 software. Results were considered significant at *P *< 0.05 (two-tailed).

The size of our sample was sufficient to detect a significant difference with a power of more than 70% assuming an OR value for AA of 1.5 with a minor allele frequency of 0.1 and type I error rate set at 0.05.

## Results

Four SNPs in the *GHRL *locus were analyzed: Leu72Met, -501A/C, -604 G/A, and -1062 G > C. As shown in Table 2, none of the genotype distributions of these four SNPs showed significant deviation from Hardy-Weinberg equilibrium, and none of the allele frequencies or the genotype distribution differed between patients and controls (*P *> 0.05). There was also no significant association between any allele or genotype and SZ when patients were subdivided by gender (Table 3).

**Table 2:** Genotype and allele frequencies of four SNPs in *GHRL *gene between schizophrenia patients and healthy controls

Marker	N ^a^	Genotype ^b^			HWE	*P*-value	Allele ^b^		*P*-value	OR^c ^(95%CI)
rs696217		GG	GT	TT			G	T		
Patients	634	427(0.684)	180(0.288)	17(0.027)	0.704	0.649	1034(0.829)	214(0.171)	0.773	1.03(0.83-1.27)
Controls	606	400(0.670)	184(0.308)	13(0.022)	0.123		984(0.824)	210(0.176)		
rs26802		AA	AC	CC			A	C		
Patients	634	534(0.842)	95(0.150)	5(0.008)	0.732	0.944	1163(0.917)	105(0.083)	0.922	1.01(0.76-1.35)
Controls	606	505(0.839)	93(0.154)	4(0.007)	0.900		1103(0.916)	101(0.084)		
rs27647		AA	AG	GG			A	G		
Patients	634	10(0.016)	133(0.210)	491(0.774)	0.773	0.410	153(0.121)	1115(0.879)	0.301	1.13(0.88-1.46)
Controls	606	5(0.008)	120(0.198)	480(0.793)	0.400		130(0.107)	1080(0.893)		
rs26311		CC	CG	GG			C	G		
Patients	634	253(0.402)	298(0.474)	78(0.124)	0.498	0.826	804(0.639)	454(0.361)	0.723	1.03(0.87-1.21)
Controls	606	234(0.387)	297(0.491)	74(0.122)	0.171		765(0.632)	445(0.368)		

^a ^Number of samples which are well genotyped

^b ^Frequencies are shown in parenthesis

^c ^Odds ratios of alleles were calculated for each reference *vs*. variant allele

**Table 3:** Genotype frequencies of the four SNPs interaction with gender

dbSNP ID	Genotype	Female			Male		
		
		Patients	Controls	p-value	Patients	Controls	p-value
rs696217	GG	224	191	0.689	203	209	0.896
	GT	93	91		87	93	
	TT	11	7		6	6	
rs26802	AA	279	247	0.306	255	258	0.723
	AC	50	41		45	52	
	CC	3	2		2	2	
rs27647	AA	4	2	0.151	6	3	0.368
	AG	77	51		56	69	
	GG	251	240		240	240	
rs26311	CC	142	118	0.358	111	116	0.876
	CG	150	139		148	158	
	GG	37	36		41	38	

To further analyze the haplotype structure in our sample, pair-wise linkage disequilibrium (LD) of the four SNPs in the control group was computed using the standardized measures *D*' and *r*^2 ^values. There was strong LD in Leu72Met and -501A/C, so haplotype analyses were performed (Table 4). However, the haplotypes constructed from two SNPs showed no significant differences between patients and controls (Table 5).

**Table 4:** Pairwise linkage disequilibrium among four SNPs in the *GHRL *gene *(D*' values is shown above and r^2 ^values below the diagonal)

	rs696217	rs26802	rs27647	rs26311
rs696217		**0.901**	0.004	0.261
rs26802	0.016		0.175	0.193
rs27647	0.000	0.000		0.116
rs26311	0.025	0.006	0.001	

**Table 5:** Estimated haplotype frequencies and case-control haplotype results

SNP	Haplotype	Frequencies		*χ^2^*	*P*-value	OR (95%CI)	Global	
						
		Cases	Controls				*χ^2^*	*P-*value
**rs696217--rs26802**	G-A	930.15(0.745)	883.02(0.742)	0.007	0.933	1.008(0.840~1.209)	0.022	0.989
	G-C	103.85(0.083)	97.98(0.082)	0.004	0.952	1.009(0.756~1.346)		
	T-A	213.85(0.171)	205.98(0.173)	0.020	0.888	0.985(0.798~1.216)		

Of the 634 patients with SZ, 380 completed the PANSS to assess psychopathological syndromes. The results revealed that there were no significant differences in PANSS score reduction among the different genotypes of the four SNPs (-604 G/A, -501A/C, Leu72Met and -1062 G > C) after 8 weeks treatment with AAPs (Table 6). However, in the responder group with the larger decreases in PANSS scores (> 50%), there was a significant association between BW and BMI increase (Table 7). The responder group exhibited significantly greater BW and BMI increases than none responders, and patients in specific AAP treatment groups with high weight gain showed greater improvements than those with low weight gain when subdivided according to drug (Table 8).

**Table 6:** Reduction of PANSS scores in patients with different GHRL genotypes ($\overset{-}{x}\pm s$)

SNP	Genotype	N	Before treatment				After 8 weeks treatment				Reduction rate (%)*
				
			Total**	P ^a^	N ^b^	G ^c^	total	P ^a^	N ^b^	G ^c^	
rs27647	GG	292	88.06 ± 21.64	24.49 ± 6.63	21.46 ± 8.00	42.11 ± 12.34	45.84 ± 12.63	10.87 ± 3.69	11.65 ± 4.85	23.32 ± 6.33	0.72 ± 0.19
	GA+AA	88	87.38 ± 23.51	23.05 ± 6.92	22.81 ± 8.28	41.52 ± 13.4	45.40 ± 10.86	10.68 ± 3.53	12.24 ± 5.05	22.48 ± 4.88	0.72 ± 0.19
	*P*		0.799	0.076	0.169	0.702	0.767	0.672	0.321	0.250	0.988
rs26802	AA	322	88.61 ± 22.47	24.35 ± 6.80	21.74 ± 8.26	42.52 ± 12.76	45.91 ± 12.47	10.90 ± 3.76	11.76 ± 4.97	23.25 ± 6.17	0.72 ± 0.19
	AC+CC	58	83.97 ± 19.33	23.09 ± 6.16	21.91 ± 7.00	38.97 ± 11.20	44.79 ± 10.85	10.43 ± 2.96	11.93 ± 4.49	22.43 ± 5.15	0.72 ± 0.18
	*P*		0.140	0.187	0.882	0.048	0.524	0.371	0.805	0.341	0.929
rs696217	GG	253	87.91 ± 22.94	24.23 ± 6.53	21.66 ± 8.36	42.02 ± 13.15	45.09 ± 11.31	10.63 ± 3.60	11.61 ± 4.70	22.85 ± 5.34	0.73 ± 0.18
	GT+TT	120	87.65 ± 19.38	24.11 ± 6.96	21.82 ± 7.36	41.73 ± 10.91	45.97 ± 11.41	11.01 ± 3.31	11.74 ± 4.70	23.22 ± 6.10	0.71 ± 0.20
	*P*		0.915	0.870	0.864	0.834	0.484	0.329	0.798	0.555	0.309
rs26311	CC	153	87.25 ± 22.59	24.09 ± 6.84	21.58 ± 7.52	41.58 ± 13.06	45.30 ± 11.10	10.85 ± 3.70	11.52 ± 4.52	22.93 ± 5.35	0.72 ± 0.19
	GG+GC	223	87.66 ± 21.04	24.09 ± 6.59	21.74 ± 8.36	41.83 ± 11.82	46.01 ± 13.04	10.79 ± 3.63	11.96 ± 5.15	23.25 ± 6.49	0.72 ± 0.19
	*P*		0.857	0.998	0.845	0.848	0.582	0.884	0.385	0.619	0.883

*reduction rates of PANSS total scores; ** total scores; ^a ^Positive score; ^b ^negative score; ^c ^general pathology score

**Table 7:** The BW and BMI change in responder group and none-responder groups

Two group ^a^	N	BW change ^b ^($\overset{-}{x}\pm s$) kg	BMI change ^c^($\overset{-}{x}\pm s$)
responder group	339	0.68 ± 4.00	0.27 ± 1.47
none-responder group	41	-1.13 ± 2.35	-0.42 ± 0.87

P-value		0.000	0.000

^a ^Patients were divided into 2 groups based on reduction rates of PANSS total scores, namely, responder group (> 50%) and none-responder group (≤ 50%)

^b ^Body weight change = body weight (4-week) - body weight(0 week)

^c ^BMI change = BMI (4-week) - BMI (0 week)

**Table 8:** The BW and BMI change in responder group and none-responder groups when subdivided according to different AAPs ($\overset{-}{x}\pm s$)

Two groups ^a^	AAP ^b^	N	BW change (kg)	P-value	BMI change	P-value
responder group	clozapine	63	0.60 ± 3.94	**0.012**	0.21 ± 1.41	**0.009**
	risperidone	115	1.20 ± 3.71		0.46 ± 1.41	
	olanzapine	25	1.94 ± 4.47		0.76 ± 1.68	
	quetiapine	76	1.01 ± 3.41		0.37 ± 1.26	
	ziprasidone	32	-1.66 ± 4.78		-0.60 ± 1.74	
	aripiprazole	26	-0.67 ± 4.42		-0.19 ± 1.49	
none-responder group	clozapine	7	-1.57 ± 2.30	0.917	-0.55 ± 0.80	0.834
	risperidone	11	-1.18 ± 1.99		-0.47 ± 0.79	
	olanzapine	5	-0.60 ± 1.82		-0.18 ± 0.62	
	quetiapine	8	-0.44 ± 2.87		-0.16 ± 1.07	
	ziprasidone	6	-1.5 ± 2.74		-0.56 ± 0.95	
	aripiprazole	3	-1.8 ± 3.69		-0.73 ± 1.42	

^a ^responder group and none-responder group

^b ^atypical antipsychotics

The main clinical and biochemical characteristics of the schizophrenic patients were analyzed with nonparametric tests. There was a significant association between BW and BMI measured before and after 4-week AAP treatment (*P *= 0.005 and 0.004 respectively). Patients with the -604 G/A exhibited significantly higher BWs and BMIs after treatment (*P *= 0.028 and 0.011, respectively) (Table 9). Similarly, paranoid SZ patients (n = 309) demonstrated greater WG and BMI increases (*P *= 0.020 and 0.011, respectively). In addition, there were significant differences in the BW and BMI increases between G allele carriers and homozygous allele A carriers in patients harboring SNP-604 G/A (*P *= 0.039 and 0.013, respectively).

**Table 9:** The association analysis of BW and BMI in four SNPs

SNP	Genotype	BW(0 week, kg)	BW(4 week, kg)	BW change (kg)	BMI(0 week)	BMI(4 week)	BMI change
rs27647	GG	62.69 ± 11.86	63.16 ± 11.19	0.55 ± 3.85	22.65 ± 3.60	22.84 ± 3.44	0.21 ± 1.42
	AG	62.85 ± 12.44	62.87 ± 11.88	-0.18 ± 3.35	22.16 ± 3.59	22.13 ± 3.48	-0.04 ± 1.18
	AA	58.4 ± 7.40	61.39 ± 10.25	2.61 ± 3.46	21.4 ± 2.91	23.05 ± 3.71	1.15 ± 1.21
	*P-*value	0.426	0.810	0.028*	0.392	0.181	0.011*
rs26802	AA	62.53 ± 12.13	62.90 ± 11.33	0.40 ± 3.79	22.54 ± 3.62	22.68 ± 3.45	0.17 ± 1.39
	AC	63.35 ± 10.90	64.40 ± 11.28	0.87 ± 3.36	22.51 ± 3.54	22.95 ± 3.55	0.30 ± 1.21
	CC	59.70 ± 9.78	56.8 ± 6.30	-2.9 ± 6.07	21.66 ± 2.03	20.68 ± 1.28	-0.97 ± 2.17
	*P-*value	0.572	0.163	0.247	0.839	0.275	0.269
rs696217	GG	62.16 ± 11.75	62.77 ± 11.29	0.62 ± 3.60	22.33 ± 3.45	22.58 ± 3.37	0.24 ± 1.30
	GT	63.57 ± 12.05	63.67 ± 11.08	0.10 ± 3.85	22.83 ± 3.57	22.86 ± 3.34	0.05 ± 1.41
	TT	63.75 ± 15.14	63.67 ± 14.83	-0.08 ± 6.49	23.60 ± 6.07	23.60 ± 6.08	0.002 ± 2.51
	*P-*value	0.446	0.589	0.319	0.358	0.676	0.254
rs26311	GG	63.30 ± 12.10	63.09 ± 10.36	0.41 ± 3.39	23.13 ± 3.74	23.12 ± 3.43	0.15 ± 1.28
	GC	62.29 ± 12.25	62.89 ± 11.32	0.49 ± 3.90	22.63 ± 3.75	22.86 ± 3.48	0.20 ± 1.43
	CC	62.77 ± 11.52	63.24 ± 11.65	0.41 ± 3.71	22.21 ± 3.34	22.39 ± 3.44	0.16 ± 1.34
	*P-*value	0.792	0.925	0.804	0.343	0.344	0.765

## Discussion

The associations between *GHRL *polymorphisms and SZ risk, changes in weight/BMI, and therapeutic responses to AAPs were evaluated in a population of SZ patients of Han Chinese ethnicity. While we found no association between *GHRL *gene polymorphisms and SZ susceptibility in this case-control study, analysis did reveal significant BW and BMI increases during AAP treatment in patients harboring the -604 G/A polymorphism.

To our knowledge, no previous study has examined the association between *GHRL *gene polymorphisms and susceptibility to SZ. Our study revealed no significant differences in allele and genotype frequency of four *GHRL *SNPs between schizophrenic patients and controls even when patients were subdivided by gender. Thus, *GHRL *is not a likely SZ risk gene despite the fact that it is in a susceptibility locus (3p25-p26). Furthermore, we also examined paranoid SZ cases in light of the study by Scassellati et al. [38]. Again, we found no significant differences in the frequency of these four SNPs or the genotype distribution between paranoid patients and controls, but this could reflect the relatively small sample size of paranoid schizophrenic patients in our cohort. Furthermore, we found no haplotypes with significantly higher frequency between cases and controls. Therefore, we suggest that *GHRL *is not a predisposing gene for SZ in the Chinese Han population.

In the present study, no association between PANSS reduction during AAP treatment and *GHRL *gene polymorphisms was found. However, the magnitude of the PANSS score reduction was significantly associated with the increase in BW and BMI during AAP treatment. Meanwhile, the reduction rate of PANSS total score in responder and none-responder groups had significant association with BW and BMI increase. The same finding was also revealed when patients were subdivided according the specific AAP taken. Atypical antipsychotics induced weight gain in a significant fraction of SZ patients [9], but factors that are predictive of weight gain during AAP therapy are unclear. We found that patients exhibiting the greatest weight gains while receiving olanzapine, risperidone, clozapine, or quetiapine also showed greater improvements in symptoms than those showing lower weight gain. This result is in partial accord with a previous study that found olanzapine-induced weight gain correlated negatively with baseline BMI and positively with clinical global improvement and the length of olanzapine treatment [39].

The *GHRL *gene may be a promising candidate underlying AP-induced weight gain [40]. We found significant differences between the three -604 G/A genotypes, with patients harboring AA showing the greatest weight gain and increase in BMI. In addition to BMI, -604 G/A has been linked to variations in blood pressure [12]. Previous studies have also reported that the Leu72Met polymorphism was significantly associated with BMI [10,11]. However, we found no association between Leu72Met polymorphisms and the AAP-induced BMI increase, consistent with previous findings [26,31,32]. In addition to the significant association between the AA genotype and BMI, we also found that paranoid SZ patients demonstrated higher weight gain than patients with other subtypes of SZ, including catatonic, collapse, residual, and undifferentiated patients. Therefore, our results provide suggestive evidence for a link between -604 G/A and metabolic syndrome in paranoid SZ.

## Conclusion

While we did not find an association between *GHRL *alleles and susceptibility to SZ in the Chinese Han population, the -604 G/A polymorphism, and particularly the AA genotype, was associated with larger increases in BW and BMI in SZ patients under treatment with AAPs. Surprisingly, patients showing the greatest weight gain also showed the greatest improvements in symptoms. In order to more precisely define the impact of antipsychotic medications on metabolic parameters, control of patient subtype, sample size, as well as monitoring of multiple metabolic indices during antipsychotic therapy are of paramount importance.

## Abbreviations

SZ: Schizophrenia; *GHRL: *Ghrelin; WG: Weight gain; AAPs: Atypical antipsychotics; BMI: Body mass index; SNPs: Single nucleotide polymorphisms; BW: Body weight; DSM-IV: Diagnostic and statistical manual of mental disorders fourth edition; PCR-RFLP: Polymerase chain reaction-based-restriction fragment length polymorphism; OR: Odds ratio; 95% CI: 95% confidence intervals; LD: Linkage disequilibrium; ANOVA: Analysis of variance; PANSS: Positive and negative symptom scale.

## Competing interests

The authors declare that they have no competing interests.

## Authors' contributions

LL, WY and DZ participated in the design of the study and made final approval of the version to be published. YY were involved in drafting the manuscript and data analysis. WL, YY, HY, HZ, XS, BX, and GY carried out the molecular genetic examination. YY, WY, CJ, and JZ conducted sample selection and data management. All authors read and approved the final manuscript.
